# Magnetic resonance imaging of vulvar dermatofibrosarcoma protuberans – report of a case

**DOI:** 10.2478/raon-2013-0039

**Published:** 2013-07-30

**Authors:** Evrim Ozmen, Güven Güney, Oktay Algin

**Affiliations:** 1 Department of Radiology; 2 Department of Pathology, Ataturk Training and Research Hospital, Bilkent, Ankara, Turkey

**Keywords:** dermatofibrosarcoma protuberans, imaging features, MRI, vulvar sarcoma, vulvar carcinoma

## Abstract

**Background:**

Dermatofibrosarcoma protuberans (DFSP) of the vulva is a rare low-grade soft tissue sarcoma. Magnetic resonance imaging (MRI) findings of vulvar DFSP were essentially unreported in the literature.

**Case report:**

We report a DFSP of vulva with its clinical, histological and MRI features. As far we know this is the first case of histologically confirmed vulvar DFSP presenting with MR images. The diagnosis of DFSP is usually made by histopathologic and clinical findings.

**Conclusions:**

MRI is useful both for the diagnosis of DFSP and following up the patients since it has high soft tissue resolution and no risk of radiation exposure. With MRI the relation to the adjacent anatomical structures, extension and depth of the tumour and possible lymph node involvement can also be demonstrated.

## Introduction

Dermatofibrosarcoma protuberans (DFSP) is a rare low-grade soft tissue sarcoma that occurs in dermis and usually invades the subcutaneous tissue and muscles.^1^ The incidence of DFSP is approximately 0.1% of all tissue cancers and 5–6% of all soft tissue sarcomas.^2^,^3^ As far as we know only 28 cases with vulvar DFSP were reported.^2^,^3^ However, detailed magnetic resonance imaging (MRI) findings of vulvar DFSP were essentially unreported in the literature.^1^ Here in we report a DFSP of vulva with its clinical, pathological and MRI features.

## Case report

**FIGURE 1.**
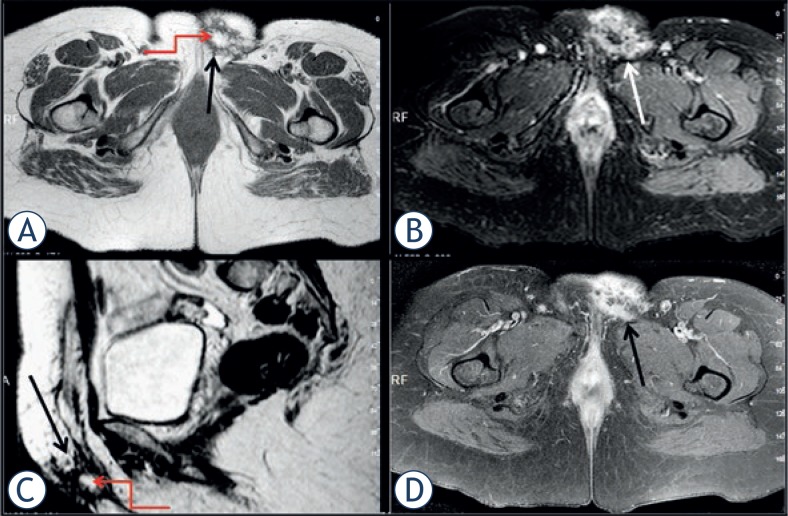
Axial T1W **A.**, axial STIR **B.**, sagittal T2W **C.**, and axial contrast enhanced T1W **D.** images. A lesion located at the vulva with irregular contours and spicular extension to the adjacent subcutaneous fat tissue (arrows). Lipomatous area at the centre of lesion (curved arrows **A.** and **C.**). Multiple foci with variable signal characteristics and fibrotic changes around the lesion. Marked contrast-material enhancement of the lesion after intravenous administration of the contrast media (arrow, **D.**).

A 60-year-old woman complaining of slowly growing foci of nodular mass extending beyond left vulva towards the left groin was admitted to our hospital. Physical examination revealed an approximately 6 cm erythematous plaque. MRI including T1 and T2 weighted (W), short tau inversion recovery (STIR) and contrast enhanced fat-suppressed T1W sequences were performed. On T1W sequence, the lesion was hypo intense; on T2W and STIR sequences it was hyper intense and after the administration of contrast-material it was enhanced heterogeneously (Figure 1). There was a lipomatous area at the centre of the lesion on all of the sequences. The contour of the lesion was irregular with spicular extension to the adjacent fat tissue. The lesion was limited with the subcutaneous fat tissue.

**FIGURE 2.**
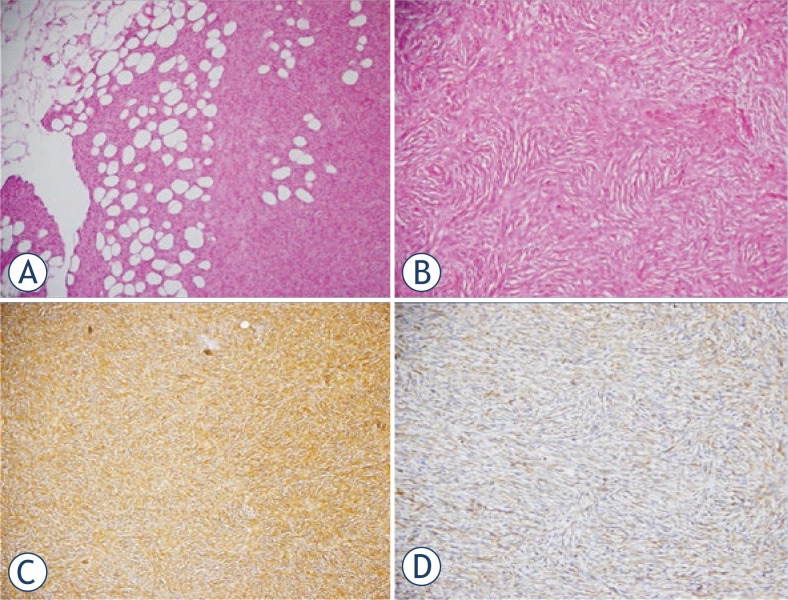
Photomicrographs of the patient. Honeycomb pattern interdigitates with lobules of subcutaneous fat (H and E, ×100) **A.** Uniform population of slender fibroblasts arranged in storiform pattern (H and E, ×200) **B.** Diffuse cytoplasmic CD34 (C; H and E, ×100) and vimentin (D; H and E, ×200) positivity **C., D.**

**FIGURE 3.**
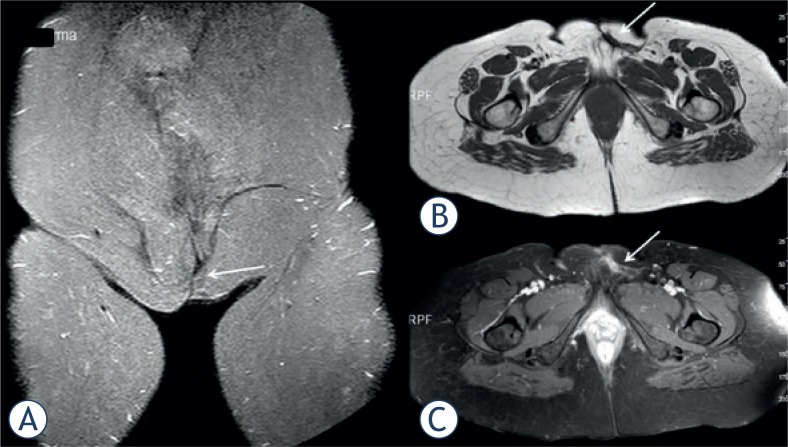
Coronal STIR **A.**, axial T1W **B.**, and axial contrast enhanced T1W **C.** MR images obtained 2 years after the surgery: postoperative changes in the operation area (arrows), no sign detected related to the recurrence.

The patient was operated. Wide resection was performed and rectus abdominis muscle-skin flap was adapted to the defect area. Histologic examination revealed a tumour specimen in a storiform and honeycomb pattern which was stained positive for CD 34 and vimentin indicating DFSP (Figure 2). The patient was followed up by regular dressing and she was discharged with recommendations. At the control of the second year the patient had no complaint. Control MRI images demonstrated post-operative changes and there was no MRI signs of illness relapse (Figure 3).

## Discussion

DFSP is a rare low grade soft tissue malignancy and is characterized by local invasion and recurrence.^1^ Males and females are affected equally.^1^–^3^ Although DFSP can occur in all ages, it most commonly occurs in young and middle aged people especially in fourth decade.^2^ DFSP may involve any part of the body but is mostly seen in trunk (42–62%).^1^–^3^ It occurs on extremities in 16% to 30% of cases, followed by head and neck (10–16%).^3^ Involvement of the vulva is extremely rare.^1^,^2^

American Musculoskeletal Tumour Society set a staging system considering the tumour grade and compartment.^4^ According to this system; stage IA represents the tumours that are low-grade intra-compartmental lesions without involvement of subcutaneous compartment. In stage IB, tumours are low-grade extra-compartmental lesions which extend beyond to the underlying fascia, muscle, or bone.^4^ According to German Guidelines for DFSP; stage I represents locally invasive primary tumour, stage II indicates regional lymph node metastases and stage III describes distant metastases.^5^ In our patient DFSP was stage IA according to American Musculoskeletal Tumour Society staging system as we, myxoid, giant cell angiofibroma, palisaded (reminiscent of schwannoma), atrophic, fibrosarcomatoid, mixed, granular cell and sclerosing type.^6^,^7^ DFSP is composed of spindled cells in a storiform pattern and infiltrates the surrounding subcutaneous fat. It stains strongly with CD 34 and vimentin as seen on immune-histochemical studies in our case.^3^,^8^

DFSP may be asymptomatic with its characteristic clinical appearance as an irregular flesh-coloured, reddish-brown to bluish nodule or violaceous plaque. It usually occurs as a solitary lesion but multiple foci could be seen.^3^,^8^ In our patient, there were multiple primary lesions that extend between the left vulva and left groin with total size of 6 cm.

DFSP could dedifferentiate to the high grade sarcomas with an increased risk of local recurrence and metastasis. Advanced age, high mitotic index and increased cellularity are associated with poor outcome as well.^3^ Our patient had a history of recurrences but had no metastases.

Ultrasonography (US) or computed tomography (CT) could demonstrate DFSP as a heterogeneous subcutaneous solid mass with spiculated or lobulated margins.^2^,^4^,^5^,^9^ MRI can show the extension and depth of the lesion with its relation to the adjacent structures.^1^,^9^ The impact of MRI in DFSP was first described by Kransdorf in 1994.^9^ Up to now, Kransdorf and Meis-Kindbom reported eleven cases and Torreggiani reported another ten patients with MR imaging findings.^1^,^9^ However, to the best of our knowledge in the literature there is only one DFSP case of the vulva mentioned with MR examination but without added MR image or mentioned MRI features of the lesion in the report.^10^

DFSP is frequently iso intense or hypo intense to muscle on T1W images, and iso intense or hyper intense on T2W sequences. The margin of the lesion is best seen on short tau inversion recovery (STIR) sequences. On contrast-material enhanced images, it enhances heterogeneously due to the foci of haemorrhage, myxoid change, and tumour necrosis.^1^,^10^ We detected an intensive heterogeneous contrast enhancement on fat suppressed contrast-material enhanced T1W images consistent with the literature. This finding may indicate the hyper vascularity of tumour.

All of the preoperative MR sequences of the presented patient demonstrated that the contour of the lesion was irregular and there were some multiple millimetres’ foci with variable signal characteristics. We believed that these foci represented the haemorrhage, myxoid change, and/or tumour necrosis as reported in the literature. Besides, the existence of lipomatous area at the centre of the lesion could be important in the diagnosis of DFSP as in our case. All of these mentioned MRI findings which were not clearly indicated in literature could be useful in differentiating DFSP from the other soft tissue sarcomas, but however it can overlap.^1^,^9^

DFSP can also be distinguished from other soft tissue since it is generally a large-sized tumour, it has suspicious deeper component, and it has history of recurrent lesion and recurrent excision with positive surgical margins.^3^,^7^,^10^

DFSP of our patient had the characteristic clinical appearance and it was diagnosed by histological examinations after the patient was operated. Thus, we performed only MRI after the physical examination since US or CT was unnecessary for our patient.

A wide surgical resection with a margin of 2–3 cm of normal tissue is recommended as the optimal treatment for both primary and recurrent DFSP. Mohs micrographic surgery has presented as an alternative approach as well. Wide surgical resection with skin-muscle flapping was preferred for our patient.

## Conclusions

DFSP of vulva is a rare low grade soft tissue sarcoma. The diagnosis is usually made by histological examinations after suspecting clinical findings. Radiologic examinations are necessary in some conditions including large tumour size, suspicious deeper component, recurrent tumours, critical locations and recurrent excision of DFSPs with positive surgical margins. MR is the most appropriate radiologic methods since it has good soft tissue contrast resolution can demonstrate the relation to the adjacent anatomical structures, extension and depth of the tumour and show the possible lymph node involvement. Also it is useful as a follow up method.
